# Adhesion Properties and Stability of Polar Polymers Treated by Air Atmospheric Pressure Plasma

**DOI:** 10.3390/polym16111552

**Published:** 2024-05-30

**Authors:** Roxana Ciobanu, Ilarion Mihăilă, Cătălin Borcia, Gabriela Borcia

**Affiliations:** 1 Iasi Plasma Advanced Research Center (IPARC), Faculty of Physics, Alexandru Ioan Cuza University, Blvd. Carol I No. 11, 700506 Iasi, Romania; 2 Integrated Center of Environmental Science Studies in the North-Eastern Development Region (CERNESIM), Alexandru Ioan Cuza University of Iasi, Blvd. Carol I No. 11, 700506 Iasi, Romania

**Keywords:** plasma treatment of polymers, atmospheric pressure plasma, polyethylene terephthalate, polyetheretherketone, polymethyl methacrylate, adhesion, contact angle, XPS

## Abstract

This study continues the discussion on the surface modification of polymers using an atmospheric pressure plasma (APP) reactor in air. These results complement prior research focusing on nonpolar polymers. Polymers, such as polyethylene terephthalate, polyetheretherketone, and polymethyl methacrylate, containing structurally bonded oxygen are studied, representing a range of properties such as oxygen content, crystalline/amorphous structure, polarity, functionality, and aliphatic/aromatic structure. APP induces superior wetting properties on the hydrophilic polymer surfaces with rapid and uniform modification within 0.5 s of exposure. The amorphous structures undergo additional modification for longer exposure. Moreover, the aliphatic chain structures require longer plasma exposure to reach surface modification equilibrium. The polar polymers reach a limit level of modification corresponding to a minimum water contact angle of about 50°. The surface polarity increases on average by a factor of approximately two. The equilibrium values of the adhesion work attained after post-processing recovery fall within a limited range of about 100–120 mJ/m^2^. The enhancement of surface functionality through the creation of oxidized groups primarily depends on the initial oxygen content and reaches a limit of about 40 at.% oxygen. The surface properties of the treated polar surfaces exhibit good stability, comparable to that of the previously tested nonpolar polymers.

## 1. Introduction

Polymers, as ubiquitous materials, offer a large array of bulk characteristics, particularly versatile mechanical properties, making them indispensable across all sectors. Their wide-ranging bulk properties result from the complex combination of chemical composition, chain structure, the presence of functional groups, and crystalline/amorphous character [1]. However, their surface properties are critical in numerous applications. Polymer surfaces typically exhibit hydrophobicity and intrinsic inertness, which raise challenges in applications requiring convenient adhesion, friction, penetrability, wettability, dyeability, and biocompatibility. Therefore, it is essential to employ appropriate methods and techniques to tailor the surface of polymer materials to meet the requirements of specific applications [2,3,4,5,6,7,8,9,10,11].

At present, the advancement of surface processing techniques must adhere to the general guidelines and recommendations advising the use of innovative technologies with reduced environmental impact. These technologies should facilitate the production and tuning of materials with enhanced durability. In this context, plasma technologies have emerged as valuable tools for the surface processing of polymers. The rapid, clean, and environmentally friendly plasma-based processes can induce physical and chemical changes in polymers in the topmost surface, generally preserving the original bulk qualities [12,13,14,15,16]. Moreover, atmospheric pressure plasmas (APPs) are particularly advantageous for activating or modifying polymer surfaces. They offer benefits such as eliminating the engineering costs associated with low-pressure plasmas and reducing the environmental impact of harmful by-products [17,18].

However, employing APP for the surface processing of polymers needs to address two primary challenges. Firstly, the surface treatment must yield stable surface properties, minimizing the recovery effect on plasma-exposed surfaces [17,19]. Secondly, the density of plasma active species (O^•^, OH^•^, etc.) needs to be sufficiently high to ensure rapid, efficient, and uniform surface treatment [13]. In this context, the benefits of utilizing plasmas generated in filamentary mode-operated discharges should be considered. A stable filamentary discharge mode is less susceptible to minor changes in electrode configuration or small variations in the amplitude or repetition frequency of the applied voltage. Additionally, it provides an excellent source of microdischarges containing energetic electrons. The electron energies, typically ranging from 1 to 10 eV, due to the reduced field at the breakdown in most gases, fall within the ideal range for exciting atomic and molecular species and generating radicals through the breaking of chemical bonds [16,20,21,22].

Taking this into account, we continue here the discussion on the surface modification of polymers using a plasma reactor operating in air at atmospheric pressure. The plasma is generated through a dielectric barrier discharge (DBD) of the random filamentary type, enabling the surface treatment of materials over a larger exposed area, under conditions simulating continuous processing.

The results presented here complement those previously published herein [23], which focused on nonpolar polymers. At this time, three types of polymer films containing structurally bonded oxygen are subjected to plasma treatment. These materials are selected aiming to assess the plasma’s ability to modify the surface properties of complex polymer structures and induce additional oxidation on materials with varying oxygen content. Furthermore, the study aims to quantify the extent and limitations of surface modification concerning the polymer structure and surface properties of polar oxygen-bearing polymers.

Thus, the tested polymers include polyethylene terephthalate (PET), polyetheretherketone (PEEK), and polymethyl methacrylate (PMMA), representing a range of properties such as oxygen content, crystalline/amorphous structure, polarity, functionality, and aliphatic/aromatic structure. Aromatic polymer structures typically exhibit intrinsic rigidity and chemical inertness due to the aromatic ring, resulting in enhanced chemical stability against plasma-induced modification compared to aliphatic structures. Additionally, amorphous polymer structures may undergo a higher rate of modification in a reactive environment compared to semicrystalline ones. Also, polar oxygen-bearing polymer structures may demonstrate a limited increase in their surface adhesion-related properties and an accelerated tendency to post-treatment recovery [5,24,25,26,27,28,29,30,31,32,33,34,35,36,37]. Considering these factors, this study aims to explore the complex relationship between these structural parameters and the extent and limitations of plasma-induced surface modification compared to nonpolar polymers [23].

The surface of the polymers is analyzed before and after processing using contact angle measurement (CA) and X-ray photoelectron spectroscopy (XPS). X-ray diffractograms (XRDs) are employed to analyze the amorphous/crystalline structure of the polymers and determine the degree of the crystallinity of the polymers where applicable. Furthermore, the stability of the surface properties is evaluated by contact angle measurement over a period of two weeks following treatment.

Our findings indicate that the adhesion-related properties of the polar functional polymers containing intrinsically bound oxygen can be significantly enhanced with short exposures to APP operating in the air. This highlights the particular efficiency of the APP reactor developed in our laboratory for the surface treatment of the polymers. The APP treatment results in consistent surface modification within very short plasma exposure times for both nonpolar and polar polymers, with very good stability of the surface properties. In this context, most results reported in the literature involve significantly longer exposure durations, usually at least 5 s and up to several minutes, with many studies either not assessing stability or reporting severe ageing effects. For example, in [28], the air plasma treatment of PET results in a WCA of 12° after 1 min of exposure, which increases to 21° after 3 min, indicating a reversal of the plasma effect due to the ablation of oxidized groups. Since the stability was not tested, this may suggest surface degradation. In [26], PET treated with an APP jet for 0.5 s reaches a minimum WCA of approximately 47° at a single spot in the center of the exposed area; values as low as ~26° are achieved over more extensive areas after 5–10 min of exposure. Similarly, in [25], a WCA of 40° is reached for PET at a single spot after 10 s of exposure. In [31], plasma-treated PMMA achieves a WCA of ~50° with various gas combinations, with the lowest values obtained only upon adding oxygen to the discharge and a treatment time of 2 min. In [29], PMMA reaches a minimum WCA of 54° after 1 min of exposure. In [35], 1-min plasma-treated PEEK was reported to achieve a contact angle as low as 7°, but complete recovery to the untreated state occurred after two weeks of ageing. The same group reported severe recovery for 30 s plasma-treated PET. The hydrophobic recovery of plasma-treated PEEK is also analyzed in [33].

The results obtained in this study complement our first series of experiments, revealing that the extent of surface modification in terms of oxygen content primarily depends on the initial oxygen content of the polymer structure. Importantly, the stability of the treated polar surfaces is comparable to that of the previously tested nonpolar polymers, with the limited loss of the hydrophilic character imparted by plasma exposure. Additionally, comparing the two types of polymers reveals that the enhancement of the adhesion-related surface properties is less pronounced for the polar polymers than for the nonpolar ones. This is due to the presence of highly polar oxidized structures in the polar polymers, which limit the extent of possible surface modification. Moreover, the differing behaviors of the tested polymers emphasize the importance of assessing each specific combination of plasma, polymer, and treatment parameters. A general trend may not be applicable in all cases, and extended exposure may be required for certain polar polymers.

These findings serve to supplement previous research, advancing the design and development of APP technology for surface processing [23]. The filamentary-mode plasma generated in this study proves to be an efficient source of microdischarges, which sweep over the exposed material at a controlled speed, enabling continuous processing. The polymer surfaces undergo significant changes in their properties for very short plasma exposure. The selection of materials in this study represents various polymer classes that contain intrinsically structurally bonded oxygen in their structures prior to treatment. This allows for the determination of the achievable limit of surface modification in terms of hydrophilicity, adhesion, surface polarity, and oxygen uptake. Additionally, it facilitates comparisons between the effects of plasma treatment on amorphous polymer structures versus semicrystalline ones, as well as between aliphatic chain structures and aromatic ones.

These results also contribute to the broader context of polymer applications, where suitable adhesion-related characteristics are essential, necessitating appropriate surface treatment methods which preserve the bulk properties and allow controlling the post-processing recovery.

## 2. Materials and Methods

### 2.1. Experimental Setup

The plasma used for the surface treatment is generated using an in-house designed and built plasma reactor operating in air at atmospheric pressure, enabling exposure over a large area under conditions simulating continuous processing.

The setup of the dielectric barrier type has been previously described [23]. It consists of an asymmetrical electrode configuration with an adjustable interelectrode gap. The high-voltage electrode is a double-blade system, 20 cm long and 2 mm wide. The sample to be treated is positioned on the ground electrode, which is a rectangular metallic plate, 30 cm × 20 cm, covered with a dielectric layer (polymer film). During the treatment, a motor stage moves the ground electrode, enabling the samples to pass through the discharge region between the electrodes at a linear controlled speed ranging from 0.3 to 3 cm/s. This arrangement permits the control of very short treatment times (fractions of a second). The high-voltage source consists of an electrical circuit including a trigger signal from a function generator, a high-voltage DC supply (Technix SR Series 30 kV, 20 mA, 600 W) (Technix, Créteil, France), and a solid-state electronic switching device (Behlke HTS 300, 30 kV DC, 30 A peak, rise time < 10 ns). This circuit generates high-voltage pulses, with duration in the range of hundreds of nanoseconds and with very short rise time (<100 ns). 

In these experiments, the electrical parameters are kept constant: 9.5 kV amplitude of the pulses, 40 μs width, and 900 Hz repetition frequency, generating 5 A peak current intensity and resulting in 1.4 mJ/pulse energy transferred to the discharge.

In these conditions, the plasma reactor operates in the filamentary mode, where the continuous electrical breakdown occurs through a large number of microdischarges spanning the interelectrode gap. These short-lived microdischarges are randomly distributed throughout the interelectrode zone, continuously sweeping the polymer sample. This results in the effective uniformity of the treatment across test surfaces measuring approximately 10 cm × 10 cm. 

### 2.2. Materials

**Figure 1:**
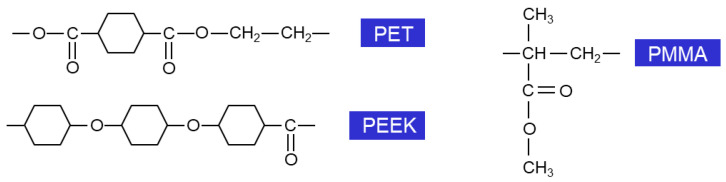
Chemical structure of the repeat units of the tested polymers (theoretical initial oxygen content: PET—40 at.%, PEEK—15.8 at.%, and PMMA—40 at.%).

The experiments are carried out on three types of commercial polymer films (Goodfellow Ltd., Cambridge, UK), 0.05 mm thick, selected to encompass a range of properties in terms of the oxygen content, crystalline/amorphous structure, polarity, functionality, and aliphatic/aromatic structure. The materials tested are polyethylene terephthalate (PET), polyetheretherketone (PEEK), and polymethyl methacrylate (PMMA). The chemical structures of the repeat units of these polymer materials are presented in Figure 1. 

The plasma exposure effects are studied for two treatment durations, 0.5 s and 1.0 s.

### 2.3. Surface Analysis

The crystalline structure of the polymer films was investigated by X-ray diffraction (XRD) using a Shimadzu LabX D6000 X-ray diffractometer (Shimadzu, Kyoto, Japan), with Cu-K_α_ X-ray source (λ = 1.54059 Å) in Bragg/Brentano configuration. The samples were scanned in the 2θ = 10°–80° range, at a 4°/min scanning rate and 2° grazing incidence. The diffraction patterns showed peaks, associated with the diffraction on the crystalline phase, superimposed on an amorphous halo. These patterns were fitted with mixed Gaussian/Lorentzian functions, with a mixing ratio > 0.8 and linear-type background subtraction. The degree of crystallinity X_c_ calculated from the ratios of the areas under the crystalline peaks A_c_ and the amorphous halo A_a_, per [38], is

X_c_ = A_c_/(A_c_ + A_a_) (1)

The contact angle measurement is carried out by the sessile drop technique using an automated system to store the drop images via an Optika 4083.B5 digital camera (Optika, Ponteranica, Italy) with PC-based control, acquisition, and data processing. The measurements were conducted under ambient conditions, with room temperature ranging between 19 and 21°. The values of the static contact angle presented are the average of at least 10 measured values obtained on the imaged sessile liquid drop profile, with a drop size of 1 μL.

Then, the water adhesion work on treated surfaces is calculated, defined as

W_a_ = γ_L_(1 + cos θ), (2)

where θ is the contact angle, and γ_L_ is the surface tension of the test liquid, water (W) or formamide (F), presented in Table 1.

The relative increase in the adhesion work, defined as

ΔW_a_/W_a_ = (W_a(treated)_ − W_a(untreated)_)/W_a(untreated)_ × 100%, (3)

is also used as a control parameter for the plasma-induced effect on the polymer surface.

Furthermore, the values of the components in Table 1 are used to calculate the surface energy (γ_S_) of the polymer sample and its polar (γ_S_^p^) and dispersive (γ_S_^d^) components, using the Owens, Wendt, Rabel, and Kaelble (OWRK) model [39,40].

Then, the total surface energy of the polymer sample is

γ_S_ = γ_S_^d^ + γ_S_^p^ (4)

and the surface polarity is defined as

γ_S_^p^/γ_S_ = γ_S_^p^/(γ_S_^d^ + γ_S_^p^). (5)

Furthermore, the contact angle of water (WCA) is used to monitor the surface’s ageing post-treatment. The post-treatment measurement of WCA is conducted on material strips (~8 cm × 0.5 cm) cut from a larger piece that has been exposed to plasma (~10 cm × 10 cm). The strips are then stored in sealed boxes, in a dry and cool room, throughout the ageing process. WCA is measured at various intervals up to 14 days after plasma exposure, providing a sensitive indication of the surface’s tendency to revert to its untreated state, as evidenced by an increasing WCA. These values are also used to calculate the adhesion work (W_a_) and to assess the surface’s tendency to reduce its surface energy. Limited variation in these parameters during ageing implies good stability of the surface properties.

The surface chemical analysis is performed by XPS, with the XPS spectra recorded on a PHI VersaProbe 5000 spectrometer (ULVAC-PHI, Kanagawa, Japan) using the Mg-K_α_ line (h*ν* = 1253.6 eV), at 45° take-off angle and 20 eV pass energy. The value of 285.0 eV of the hydrocarbon C1 core level is used as a calibration of the energy scale. The peak envelopes are curve-fitted using the PHI MultiPak software (ver. 9.6, Ulvac-PHI, Inc., Chikasaki, Japan), employing mixed Gauss/Lorentz component profiles with a mixing ratio >0.8 and linear-type background subtraction.

## 3. Results

### 3.1. XRD Analysis

**Figure 2:**
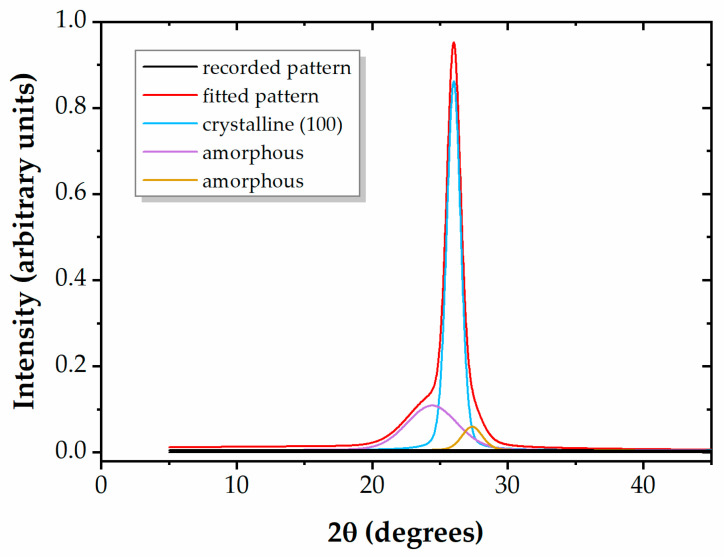
Curve-fitted diffractogram for PET.

The X-ray diffractograms highlight the completely amorphous character of the PEEK and PMMA polymers, whereas PET exhibits a distinct crystalline peak, situated at 2θ = 26.02°, as shown in Figure 2. The fitted spectrum of PET is analyzed based on reference patterns [41,42,43], and the calculation of the degree of crystallinity yields X_c_ = 0.67, indicating the pronounced crystalline structure of PET. The XRD pattern of PET remains unchanged upon plasma exposure, which is plausible since plasma affects only a few surface layers.

The differences in the amorphous/crystalline structure of the three polymers may result in variations in the treatment outcomes, because the rate of modification, driven by radical formation and subsequent reactions, is possibly higher for the amorphous polymer regions compared to the embedded crystalline regions.

### 3.2. Contact Angle Measurement

Here, all three untreated surfaces exhibit distinct hydrophilic characteristics, as illustrated by the water contact angle values (WCAs) <90°, due to the presence of polar carbon groups in their structure (as presented in Figure 1). Moreover, their initial level of wettability and polarity is similar within error bars, with WCA around 73–75°. Subsequently, WCA decreases after plasma exposure, demonstrating the plasma’s capability to induce superior wetting properties on hydrophilic polymer surfaces, as shown in Table 2. Additionally, the rate of modification of wettability is highest during the first 0.5 s of exposure, followed by limited evolution.

However, interestingly, the relative modification differs for the three polymers, despite their similar initial values. Thus, the difference between the treated and untreated surfaces ranges as (PEEK) > (PET) > (PMMA), which raises some questions regarding the correlation between the polymer structure parameters and the extent of modification. 

In the case of the semicrystalline PET, the enhancement in wettability practically levels off with prolonged exposure, whereas additional enhancement occurs for PEEK and PMMA, likely due to the higher susceptibility of the amorphous structure to undergo radical formation and functionalization during plasma processing. This trend of evolution differs from that observed with amorphous polystyrene (PS) in our previous study [23], possibly due to the combination of the amorphous polymer structure and the hydrophilic character of the surface of PEEK and PMMA compared to PS. 

It is worth noting the difference in behavior between the aliphatic chain structure of PMMA and the aromatic chain structures of PEEK and PET, where PMMA exhibits a visibly limited level of wettability modification. This result aligns with previous studies [19], suggesting a lower rate of radical formation, by hydrogen extraction, from methylene-type groups CH_x_ in the aliphatic chains and pendent groups compared to the aromatic ones. Moreover, it could indicate that an aliphatic structure like PMMA would require longer plasma exposure to reach surface modification equilibrium, achieved through a combination of surface oxidation and/or the loss of carbon by conversion to low-weight volatile fragments such as CO or CO_2_ [22]. 

This result underscores the importance of assessing each specific combination of plasma/polymer treatment parameters, as a general trend of evolution may not be applicable in all cases.

**Figure 3:**
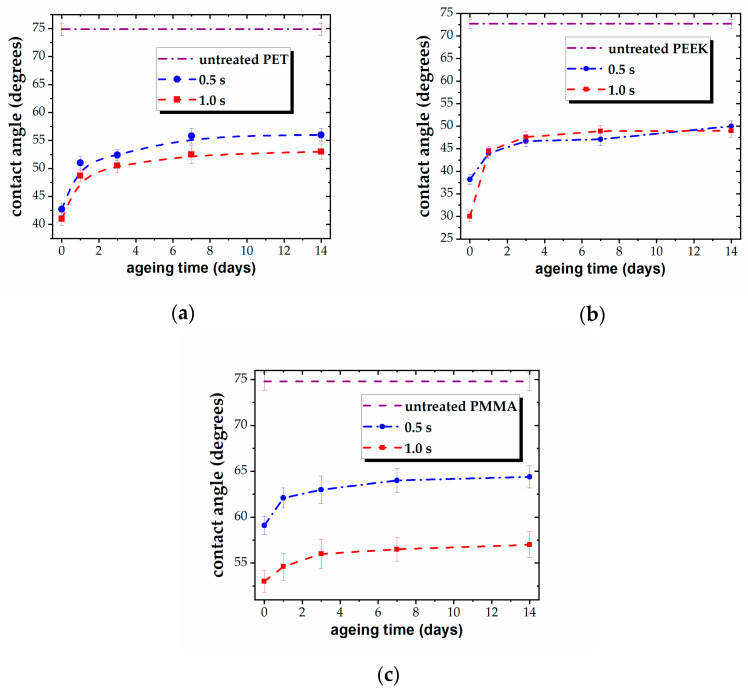
Variation in WCA vs. ageing time for plasma-treated (**a**) PET, (**b**) PEEK, and (**c**) PMMA for different treatment times (0.5 s and 1.0 s).

The different behavior of the three polymers also manifests in the post-treatment evolution of plasma-exposed surfaces. The WCA measurements taken over a two-week period after treatment indicate a more pronounced loss of the hydrophilic character in the polar polymers compared to the nonpolar ones [23], as presented in Figure 3. For PET, the WCA increases by approximately 12° for both treatment durations, whereas for PEEK, the increase is around 12° for the 0.5 s exposed surface and about 19° for the 1.0 s exposed one, further suggesting a higher reduction in wettability compared to the nonpolar polymers [23]. Conversely, for PMMA, the increase is approximately 5° for both treatment durations, implying a reduced loss in hydrophilic character, possibly due to the lower degree of surface perturbation triggered by plasma-induced increased polarity. 

For PET and PEEK, hydrophobic recovery occurs predominantly within the first 2–3 days following plasma treatment, with minor subsequent changes. Eventually, the water contact angle (WCA) values stabilize at recovery equilibrium, exhibiting no or very small differences between the values corresponding to the two exposure durations. This suggests that the polar polymers reach a limited level of modification of their surface properties, corresponding to a minimum WCA of about 50°.

In contrast, for PMMA, the recovery process is minimal and occurs primarily within the first day after treatment. Additionally, there is a distinct difference between the WCA values associated with 0.5 s and 1.0 s plasma exposures, suggesting a slower progression in plasma-induced surface modification for the aliphatic chain structure.

In the present experimental conditions, the optimal plasma treatment time appears to be 0.5 s for most tested polymers. However, extended exposure may lead to stronger hydrophilic properties for certain polar polymers, as observed in the case of PMMA.

Importantly, all three polymers display enhanced wettability after post-treatment recovery compared to their untreated surfaces.

It is important to note that the wettability properties of the treated polymers, assessed through WCA measurements, are primarily related to surface chemistry. While surface morphology was not examined in this study, previous research on various polymers treated with different atmospheric pressure DBD arrangements has demonstrated that changes in average surface roughness are minimal, typically only a few nanometers. Therefore, the influence of surface roughness on the measured contact angle is likely negligible [23,44,45,46].

The improvement of the adhesion properties in the plasma-treated polar polymers is illustrated by the increase in water adhesion work (W_a_), calculated using Equation (2), and its relative variation (ΔW_a_/W_a_,) calculated using Equation (3), as presented in Table 3.

The values presented in Table 3 confirm that the three polymers exhibit similar adhesion properties before treatment. After post-treatment recovery, PET and PEEK show a comparable enhancement in their adhesion properties, while PMMA demonstrates more limited improvement.

The relative variation ΔW_a_/W_a_ on plasma-exposed polymers appears to be lower for the polar structures examined here compared to the previously tested nonpolar materials [23]. This is because the adhesion work is calculated using Equation (2), where higher values of the contact angle θ result in a greater variation in W_a_. Therefore, the modification of hydrophobic surfaces (WCA > 90°) appears more pronounced in terms of adhesion properties compared to the polar hydrophilic surfaces (WCA < 90°). Due to the same equation, the significant difference in contact angle between the PEEK surfaces exposed to plasma for various durations seems only moderate in terms of adhesion work. 

Nonetheless, one should note the measurable improvement in wettability and adhesion-related properties for polar polymer structures exposed to plasma for such short durations, as well as the maximum level of modification achievable using the plasma treatment. In this regard, the maximum values for W_a_, which were around 110 mJ/m^2^ for pentru polyethylenes (PEs) and polypropylene (PP), and approximately 128 mJ/m^2^ for polystyrene (PS) [23], are roughly 128 mJ/m^2^ for PET, 136 mJ/m^2^ for PEEK, and 117 mJ/m^2^ for PMMA. After reaching the post-treatment recovery equilibrium, the maximum values were, in the case of the nonpolar polymers, about 103 mJ/m^2^ for PEs and PP, and around 113 mJ/m^2^ for PS [23], while for the polar polymers, they are approximately 117 mJ/m^2^ for PET, 120 mJ/m^2^ for PEEK, and 112 mJ/m^2^ for PMMA. It is evident that the rate of recovery varies for different polymer structures. However, the equilibrium values are situated in a rather limited range of about 100–120 mJ/m^2^.

**Figure 4:**
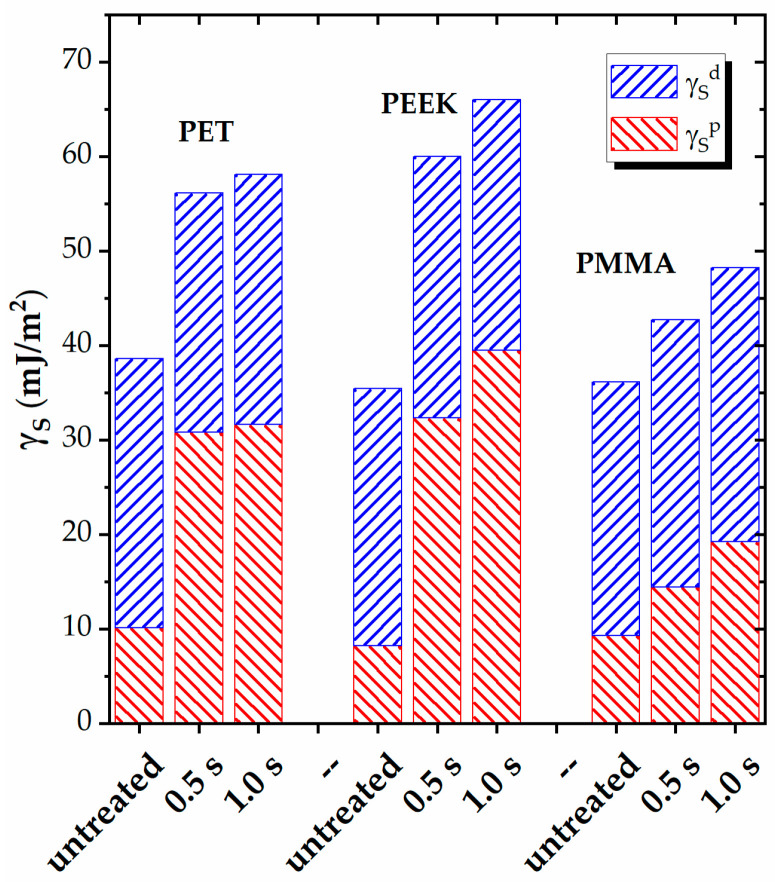
Polar (γ_S_^p^) and dispersive (γ_S_^p^) contributions to the surface energy (γ_S_) of PET, PEEK, and PMMA before and after the plasma treatment for different durations (0.5 s and 1.0 s).

In addition to the adhesion work, which provides an overall assessment of the adhesion properties, the calculation of the surface free energy (γ_S_) and its polar (γ_S_^p^) and dispersive (γ_S_^d^) components, before and after the plasma exposure, enables an evaluation of the incorporation of the polar groups onto the surface and the increase in surface polarity. The results for the three polymers are presented in Figure 4.

It is evident that the three polymers, which contain polar functional groups in their structure, exhibit significant polarity before the plasma treatment, with similar values for both components of the surface energy. The polar component (γ_S_^p^) ranges from approximately 8 to 10 mJ/m^2^, while the dispersive component (γ_S_^p^) ranges from approximately 26 to 28 mJ/m^2^. These values align with the comparable WCA values presented in Table 2 and are consistent with typical data for pristine polymers [47]. Consequently, the surface polarity γ_S_^p^/γ_S_ for the tested materials is also similar, ranging from approximately 0.23 to 0.26, as shown in Table 4.

The plasma exposure results in a measurable increase in the polar component of the surface energy for all the polymers, while the dispersive component remains relatively unchanged. The most significant increase in γ_S_^p^ is observed for PEEK, which reaches a peak value of approximately 39 mJ/m^2^ for 1.0 s plasma exposure, up from around 32 mJ/m^2^ calculated for 0.5 s exposure. Following PEEK, PET shows similar values of approximately 31 mJ/m^2^ for both exposure durations. PMMA exhibits the lowest increase in the polar component, with values of about 14 mJ/m^2^ and 19 mJ/m^2^ for 0.5 s and 1.0 s of exposure, respectively. However, it is worth noting that the 19 mJ/m^2^ value is practically double that of the untreated sample. This behavior aligns with the trend observed in the parameters related to wettability and adhesion discussed previously.

The surface polarity calculated for the three plasma-treated materials, presented in Table 4, confirms these results, with PET and PEEK reaching γ_S_^p^/γ_S_ greater than 0.55, while PMMA attains only γ_S_^p^/γ_S_ = 0.40. Comparing these results to those previously reported for the nonpolar polymers, the polyolefins (PEs and PPs) demonstrated values of 0.54–0.55, and PS exhibited a higher value of 0.60 [23]. The seemingly lower level of polarity associated with the plasma-treated polar polymers is due to their higher dispersive component of the surface energy (γ_S_^d^) overall, resulting in a higher total surface energy (γ_S_) and, consequently, a lower calculated γ_S_^p^/γ_S_ ratio according to Equation (5).

These results underscore the plasma’s capability to significantly increase the surface polarity of various polymer structures and highlight the limited level of modification achievable in terms of surface properties. The enhancement in surface polarity is less pronounced for the polar polymers compared to the nonpolar ones because the former already contain highly polar oxidized structures prior to treatment, limiting the extent of surface modification possible.

### 3.3. Surface Chemical Characterization by XPS

XPS analysis was employed to investigate the chemical structure of the polymer surfaces, focusing on the level of oxidation induced in the treated samples, to explore the correlation between enhanced hydrophilic character and increased surface polarity with augmented surface functionality.

It is important to note that the tested materials exhibit highly oxidized structural features before the plasma treatment, as shown in the chemical structure of their repeat units (Figure 1). Therefore, the XPS analysis considers that the same carbon-bonded-to-oxygen groups are likely present in the C1 spectra of both the untreated and treated polymers (for two different exposure durations). Although new groups could also be detected, the primary modification likely involved changes in the relative intensity of the peaks within the C1 spectrum. 

The XPS spectra were fitted based on reference measurements, with carbon chemical groups identified and numbered in the increasing order of their binding energy [48].

The PET, PEEK, and PMMA samples display C1 spectra fitted with three components, which are not identical for all three materials, each assigned to specific carbon functional groups, as detailed in Table 5. These components correspond to hydrocarbon atoms (C1), carbons each singly bonded to an oxygen atom (C2), carbon atoms in the carbonyl groups (C3), and carbons in the carboxyl groups (C4).

In addition, PET and PEEK exhibit a fourth characteristic peak at around 291.8 eV (C5), attributed to low energy *π*–π* shake-up transitions accompanying the core level ionization for carbons in the aromatic ring. Notably, for these two materials, the C1 component represents both aliphatic carbons and aryl carbons present. Specifically, in PEEK, 18 out of the 19 carbons in the repeat unit are aromatic-bonded atoms, while in PET, this situation applies to 6 out of the 10 carbons.

**Figure 5:**
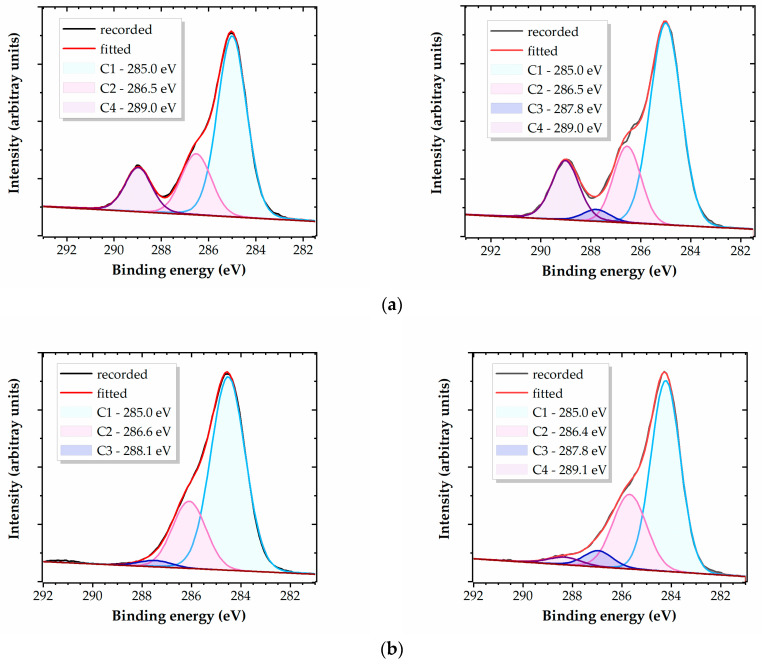

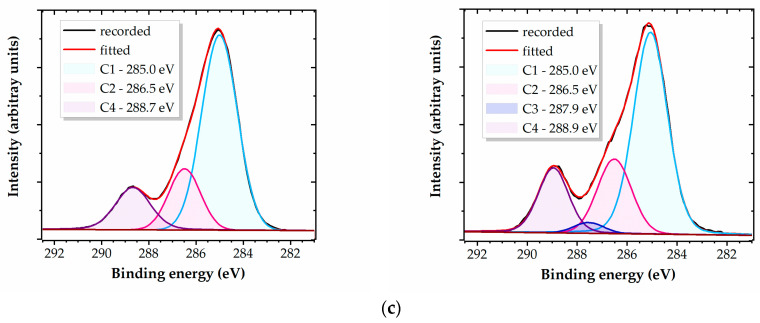
Typical deconvolutions of the high-resolution C1 XPS spectra for PET (**a**), PEEK (**b**), and PMMA (**c**), untreated (**left**) and 1.0 s plasma-treated (**right**).

Following the plasma treatment in air, the contributions of higher binding energy carbons increase, and all four peaks C1–C4 become visible on the spectra. Specifically, C3 appears for PET and PMMA, and C4 for PEEK, as shown in Figure 5.

Table 6 presents the data for the relative atomic composition of the carbon groups resulting from the deconvolution of the C1 high-resolution XPS spectra. It is important to note that the peaks provided by the carbon species linked by covalent bonds in polymers have larger widths compared to other materials, with an FWHM (full width at half maximum) of approximately 1.4–1.6 eV, leading to strong overlapping inside the C1 envelope. Hence, the separation between some functional groups may be affected by larger errors. 

Nonetheless, the presence of additionally oxidized species on the plasma-treated polymer surfaces is also demonstrated by the measurable oxygen uptake on the samples. In this respect, Table 6 includes the oxidation degree (O/C), defined here as the ratio between the oxidized and un-oxidized carbon atoms on the surface, which is also presented in Table 6 and calculated as

O/C = (C2 + C3 + C4)/C1.(6)

The capacity of our APP reactor to induce additional oxidation on such polar materials, which are carrying intrinsically bound oxygen, can be further emphasized by the data in Table 7. This table presents the total oxygen content measured on the surfaces of the untreated polymers (O_i_) and the relative variation in this content (ΔO/O_i_), calculated as

ΔO/O_i_ (%) = (O_(treated)_ − O_(untreated)_)/O_(untreated)_ × 100%. (7)

It is important to note that although the relative increase in oxygen content may appear moderate, it pertains to polymers with a significant initial oxygen content and very short plasma exposure durations. These data complement our previous findings on the plasma-treated nonpolar polymers [23], demonstrating the APP’s capability to enhance the surface functionality of oxidized structures.

The degree of modification of the polar surfaces, in terms of oxygen uptake, appears to primarily depend on the initial oxygen content of the polymer structure. For instance, PEEK, which starts with about 27 at.% oxygen, exhibits the highest degree of modification. Moreover, its oxygen content increases from 31.5 at.% O for 0.5 s exposure to 33.8 at.% O for 1.0 s exposure, indicating an evolution with treatment duration. On the other hand, PET and PMMA, starting with comparable oxygen content of around 34–36 at.%, also reach similar oxygen content levels of approximately 39–40 at.%. Although the increase is of a few percent, it signifies the presence of additional oxidized carbon groups on the surface.

Our previous findings showed that for the structures comprising solely C–H bonds (nonpolar polymers) [23], the highest degree of oxidation was about 25 at.% O. Consequently, it appears sound that the polymers initially containing more than 25 at.% structurally bonded oxygen demonstrate resistance to further oxidation, achieving limited oxygen uptake in a reactive plasma environment. Furthermore, reasonable theoretical estimates suggest a maximum C:O ratio of 1:1, corresponding to 50 at.% O, achievable through physico-chemical modification in plasma. 

In our current experimental setup, 40 at.% O likely represents the upper limit of achievable oxidation, as evidenced by the leveling off of oxygen uptake after 0.5 s exposure for both PET and PMMA. Extended treatment times may not contribute to the further formation of oxidized groups, as the balance between oxygen incorporation and the ablation of low-mass volatile fragments, such as CO or CO_2_, would restrict the amount of oxygen stably bound at the surface.

Noteworthy, the trends observed in the contact angle and the XPS data differ for PMMA. While oxidation does not progress with treatment duration, the contact angle continues to decrease with prolonged exposure. This might occur because contact angle measurements primarily probe the first monolayer at the surface, whereas XPS explores about 50 Å at the usual 45° take-off angle. Nonetheless, the wettability- and adhesion-related parameters, calculated using the contact angle, exhibit more moderate dependence on the treatment time, as illustrated in Table 3 and Figure 4.

In summary, APP treatment has the capacity to induce additional oxidation in both the aliphatic and aromatic chain structures of oxygen-containing polymers, up to a limited threshold of oxygen uptake.

## 4. Conclusions

APP exposure in ambient air results in improved wetting properties on hydrophilic polymer surfaces, with the highest rate of modification of the adhesion-related properties during the first 0.5 s of exposure. Subsequent evolution is limited. The relative modification between the treated and untreated surfaces ranges as (PEEK) > (PET) > (PMMA), indicating that the aliphatic chain structures would require longer plasma exposure to reach surface modification equilibrium.

Measurements taken over a two-week period after the treatment indicate a more pronounced loss of hydrophilic character in the polar polymers compared to the previously studied nonpolar ones, possibly related to differences in surface perturbation generated by plasma-induced increased polarity. The polar polymers appear to reach a limited level of modification of their surface properties, corresponding to a minimum water contact angle (WCA) of about 50°.

In comparison to our previous study, the maximum values of the adhesion work (W_a_) vary, and the rate of recovery differs for different polymer structures. However, the equilibrium values attained after post-processing recovery fall within a limited range of about 100–120 mJ/m^2^ for both the polar and nonpolar polymers.

Plasma exposure results in a measurable increase in the polar component of the surface energy for all the polar polymers, while the dispersive component remains relatively unchanged, resulting in an increase in the surface polarity on average by a factor of approximately two. However, this enhancement is less pronounced for the polar polymers compared to the nonpolar ones due to the presence of highly polar oxidized structures in the former, which limit the extent of possible surface modification.

The extent of oxygen uptake in the oxygen-containing structures primarily depends on the initial oxygen content of the polymer. The increase is of a few atomic percent oxygen (at.% O), signifying the creation of additional oxidized carbon groups. The upper limit reached in the present experimental setup is likely 40 at.% O due to inherent resistance to oxidation in the polymers initially containing more than 25 at.% structurally bonded oxygen. A reasonable estimation predicts a maximum of 50 at.% O achievable through physico-chemical modification in plasma.

Similar to our previous study, 0.5 s of plasma exposure appears to be the optimal treatment time for most tested polymers. However, extended exposure may lead to stronger hydrophilic properties for certain polar polymers, as observed in the case of PMMA. This result emphasizes the importance of assessing each specific combination of plasma/polymer treatment parameters, as a general trend of evolution may not be applicable in all cases.

## Figures and Tables

**Table 1:** Surface tension components of test liquids used for contact angle measurement.

Test Liquid	γ_L_ (mJ/m^2^)	γ_L_^d^ (mJ/m^2^)	γ_L_^p^ (mJ/m^2^)
Water (W)	72.8	21.8	51.0
Formamide (F)	58.2	35.1	23.1

**Table 2:** The contact angle of water (WCA) (°) measured for PET, PEEK, and PMMA on the untreated and plasma-treated surfaces for different treatment times (0.5 s and 1.0 s).

Treatment Time	PET	PEEK	PMMA
Untreated	74.9 ± 1.1	72.7 ± 1.0	74.8 ± 1.0
0.5 s	42.7 ± 1.1	38.2 ± 1.1	59.2 ± 1.2
1.0 s	41.0 ± 1.2	30.0 ± 1.2	53.0 ± 1.1

**Table 3:** Adhesion work W_a_ (mJ/m^2^) and relative variation in the adhesion work ΔW_a_/W_a_ (%) calculated for PET, PEEK, and PMMA on the untreated surfaces, plasma-treated surfaces for different treatment times (0.5 s and 1.0 s), and aged plasma-treated surfaces.

		PET	PEEK	PMMA
Untreated	W_a_ (mJ/m^2^)	91.8	94.4	91.9
0.5 s	W_a_ (mJ/m^2^)	126.3	130.0	110.2
	ΔW_a_/W_a_ (%)	38%	38%	20%
Aged 14 days	ΔW_a_/W_a_ (%)	24%	27%	14%
1.0 s	W_a_ (mJ/m^2^)	127.7	135.8	116.6
	ΔW_a_/W_a_ (%)	39%	44%	27%
Aged 14 days	ΔW_a_/W_a_ (%)	27%	28%	22%

**Table 4:** Surface polarity γ_S_^p^/γ_S_ calculated for PET, PEEK, and PMMA on untreated and plasma-treated surfaces for different treatment times (0.5 s and 1.0 s).

Treatment Time	PET	PEEK	PMMA
Untreated	0.26	0.23	0.26
0.5 s	0.54	0.54	0.34
1.0 s	0.55	0.59	0.40

**Table 5:** Binding energies of carbon functional groups in the C1 fitted spectra for the untreated PET, PEEK, and PMMA.

Functional Groups	Assignment			Binding Energy (eV)
	PET	PEEK	PMMA	
carbon/hydrogen –C–C–, –C–H	C1	C1	C1	285.0
carbon/oxygen –C–O–	C2	C2	C2	286.5 ± 0.2
carbonyl –C=O	–	C3	–	288.0 ± 0.2
carboxyl –O–C=O	C4	–	C4	289.0 ± 0.2
*π*–π* shake-up	C5	C5	–	~291.8

**Table 6:** Atomic composition of the carbon species C1 (in atom %, ±0.5 at.%) and oxidation degree O/C (± 0.03) for PET, PEEK, and PMMA on the untreated and plasma-treated surfaces for different treatment times (0.5 s and 1.0 s).

	PET			PEEK			PMMA		
	0 s	0.5 s	1.0 s	0 s	0.5 s	1.0 s	0 s	0.5 s	1.0 s
C1	64.1	59.9	59.5	73.4	68.5	66.2	66.0	61.3	60.6
C2	21.0	20.0	20.1	24.3	25.6	25.5	18.5	20.9	20.6
C3	–	3.3	3.5	2.3	4.7	6.7	–	1.6	2.3
C4	14.8	16.8	16.9	–	1.2	1.6	15.5	16.2	16.5
O/C	0.56	0.67	0.68	0.36	0.46	0.51	0.51	0.63	0.65

**Table 7:** Oxygen content for the untreated polymers O_i_ (at.%) and relative variation in the total oxygen content ΔO/O_i_ (%) for PET, PEEK, and PMMA on the plasma-treated surfaces for different treatment times (0.5 s and 1.0 s).

		PET	PEEK	PMMA
O_i_ (at.%)	untreated	35.9	26.6	34.0
ΔO/O_i_ (%)	0.5 s	12%	18%	14%
ΔO/O_i_ (%)	1.0 s	13%	27%	16%

## Data Availability

Data are contained within the article.
